# Emotional event-related potentials are larger to figures than scenes but are similarly reduced by inattention

**DOI:** 10.1186/1471-2202-13-49

**Published:** 2012-05-20

**Authors:** Henrik Nordström, Stefan Wiens

**Affiliations:** 1 Department of Psychology, Stockholm University, Stockholm, Sweden

## Abstract

**Background:**

In research on event-related potentials (ERP) to emotional pictures, greater attention to emotional than neutral stimuli (i.e., motivated attention) is commonly indexed by two difference waves between emotional and neutral stimuli: the early posterior negativity (EPN) and the late positive potential (LPP). Evidence suggests that if attention is directed away from the pictures, then the emotional effects on EPN and LPP are eliminated. However, a few studies have found residual, emotional effects on EPN and LPP. In these studies, pictures were shown at fixation, and picture composition was that of simple figures rather than that of complex scenes. Because figures elicit larger LPP than do scenes, figures might capture and hold attention more strongly than do scenes. Here, we showed negative and neutral pictures of figures and scenes and tested first, whether emotional effects are larger to figures than scenes for both EPN and LPP, and second, whether emotional effects on EPN and LPP are reduced less for unattended figures than scenes.

**Results:**

Emotional effects on EPN and LPP were larger for figures than scenes. When pictures were unattended, emotional effects on EPN increased for scenes but tended to decrease for figures, whereas emotional effects on LPP decreased similarly for figures and scenes.

**Conclusions:**

Emotional effects on EPN and LPP were larger for figures than scenes, but these effects did not resist manipulations of attention more strongly for figures than scenes. These findings imply that the emotional content captures attention more strongly for figures than scenes, but that the emotional content does not hold attention more strongly for figures than scenes.

## Background

Because sensory systems have a limited capacity, stimuli that are relevant for the survival of organisms need to be prioritized for processing. Consistent with this idea, emotional stimuli tend to attract more attention than do neutral stimuli, and this natural state of selective attention is referred to as ‘motivated attention’ [1,2]. The concept of motivated attention predicts that emotional pictures will capture attention even when the pictures are presented outside of directed attention [2-4]. To test this prediction, a common approach has been to let subjects perform a task on neutral stimuli (e.g., line bars or letters) presented in attended locations while emotional pictures are shown as distracters in unattended locations, i.e. outside of directed attention. For example, pairs of lines were presented to the left and right close to fixation while emotional or neutral faces were presented in pairs in the left and right periphery [5]. Attention was manipulated by asking participants to perform discrimination tasks either on the peripheral faces or on the central lines. This and similar designs have been used in studies recording event-related potentials (ERPs) [5-14]. In these studies, greater attention to emotional than neutral stimuli (motivated attention) was indexed with the early posterior negativity (EPN) and the late positive potential (LPP) [2-4]. These two indexes are typically obtained from difference waves between emotional and neutral stimuli; that is, the ERPs to emotional stimuli are subtracted from the ERPs to neutral stimuli. Accordingly, in the present paper, the terms EPN and LPP are used to refer to the ERP differences of emotional minus neutral stimuli. The EPN peaks around 250 ms after stimulus presentation and reflects a relative negativity over temporal-occipital electrodes to emotional (negative or positive) versus neutral stimuli. The LPP starts around 300 ms after stimulus presentation and can continue several seconds and reflects a relative positivity over central parietal electrodes to emotional versus neutral stimuli. Whereas the EPN reflects a call for attentional resources, the LPP reflects the allocation of attentional resources to salient events [2-4,13,15].

In ERP research that studied effects of inattention on EPN and LPP, emotional stimuli were fearful faces [5,7,8], emotional pictures [6,9,12-14,16,17] from the International Affective Picture System (IAPS) [18], pictures from the IAPS set together with similar pictures retrieved from the internet [10], and spiders and mushrooms [11]. The pictures were presented either in the periphery [5,6,8,9] or at fixation [6,12-14,16,17]. Most studies found that when pictures were attended, there was an EPN and LPP to emotional versus neutral pictures; however, when the pictures were unattended, emotional effects on the EPN [6,14] and LPP [5,6,8,9,14] were strongly reduced if not eliminated. Similarly, when pictures were shown in large format, emotional effects on the LPP were eliminated when participants attended neutral areas within the negative pictures [16,17]. These findings suggest that pictures need to be presented in attended locations in order to elicit an EPN and LPP to emotional versus neutral pictures.

However, a few studies found residual, emotional effects on EPN and LPP even when the pictures were unattended. Regarding the EPN, a residual EPN to unattended emotional versus neutral pictures was reported by Sand and Wiens [12] and Wiens et al. [13] for IAPS pictures. A similar effect was found by Holmes, Kiss and Eimer [7] for fearful faces^a^. Regarding the LPP, Sand and Wiens [12] and Wiens et al. [13,14] found residual, emotional effects on LPP to IAPS pictures and Norberg, Peira and Wiens [11] found an LPP to unattended pictures of spiders in spider fearful participants. Similarly, Mocaiber et al. [10] found an LPP to unattended pictures of mutilated bodies if the participants thought the pictures depicted real scenes. The residual EPN and LPP in these studies [7,10-14] may be explained by two aspects of the study design. First, because emotional pictures were shown at fixation, EPN and LPP may resist manipulations of attention. In support, emotional pictures elicit larger EPN and LPP at fixation than in the periphery [6], and even neutral pictures are more distracting at fixation than in the periphery [19].

Second, most studies that reported residual EPN and LPP apparently used pictures with simple figure-ground composition [7,10-13] rather than pictures with complex scene composition. For example, a figure picture would show a person sitting on a bench, whereas a scene picture would show a group of people interacting in a marketplace. Bradley et al. [20] showed that figures elicit larger LPP than do scenes, and the authors argued that figures might be more easily understood or mapped into memory and therefore, the emotional content in figures might capture attention more strongly than does the emotional content in scenes. However, in regards to the EPN, Bradley et al. [20] did not find an EPN to emotional versus neutral pictures, neither for figures nor for scenes. This null finding is surprising because many studies have reported evidence for an EPN to emotional versus neutral pictures [2-4,12-14]. However, Bradley et al. [20] found that an early positive peak (150–250 ms) over occipital sensors showed a main effect of picture composition in that across emotional and neutral pictures, amplitudes were more positive to scenes than figures. Because picture composition had an effect that resembled the EPN in terms of timing but had a mirrored topography (i.e., positivity over occipital electrodes), the authors cautioned that picture composition might confound the EPN. Consistent with this argument, Wiens, Sand and Olofsson [15] showed in an item analysis of ERP responses to 375 negative to neutral IAPS pictures that picture composition distorts the measurement of the EPN if the negative pictures are mainly scenes and the neutral pictures are mainly figures. Specifically, for negative scenes and neutral figures, emotion elicits a relative negativity between the negative scenes and neutral figures, but this effect is counteracted by a concurrent, nonemotional effect of picture composition that produces a relative positivity over the same electrodes (i.e., increases the P2) between the negative scenes and neutral figures. So, if picture composition has a stronger effect than emotion, the mean amplitudes over occipital electrodes would be more positive for negative scenes than neutral figures and thus, suggest an apparent reversed EPN. Results by Van Strien, Franken and Huijding [21] support this argument. When spider fearful and nonfearful participants viewed spiders (figures), negative IAPS pictures (apparently mainly scenes), and neutral IAPS pictures (apparently mainly figures), participants in general showed a reverse EPN to negative versus neutral IAPS. However, spider fearful participants showed an EPN to spiders versus neutral IAPS pictures, consistent with previous reports of an EPN to emotional versus neutral pictures after controlling for picture composition [4,12,15].

These findings suggest that the EPN is not only sensitive to effects of emotion [2-4,12-15] but is also affected by picture composition [15,20]. However, it has yet to be shown that the EPN to emotional versus neutral pictures is stronger for figures than scenes. Such results would extend the findings that the LPP is stronger to emotional figures than scenes and support that emotional figures capture attention more strongly than do emotional scenes [20]. Therefore, one purpose of the present study was to test whether EPN as well as LPP to emotional versus neutral pictures are larger for figures than scenes.

Further, if it can be shown that emotional figures capture attention more strongly than do emotional scenes, then figures may also hold attention more strongly than do scenes. There is tentative support for this idea [12-14]. In these studies, negative and neutral IAPS pictures were presented at fixation while participants performed a detection task either on the pictures or on letters that either surrounded the pictures [12,14] or were superimposed on the pictures [13]. Two studies used mainly figures [12,13] whereas Wiens et al. [14] used a mixture of figures and scenes. All studies showed strong EPN and LPP to negative versus neutral pictures when pictures were attended. Critically, when pictures were unattended rather than attended, the EPN was unaffected in the studies with figures [12,13] but decreased strongly in the study with both figures and scenes [14]. Effect sizes of the task by emotion interaction were *η*_*p*_^*2*^ = .04 [12], *η*_*p*_^*2*^ = .09 [13], and *η*_*p*_^*2*^ = .40 [14]. Similarly, the LPP apparently decreased less in the studies with figures than in the study with both figures and scenes. Effect sizes of the task by emotion interaction were *η*_*p*_^*2*^ = .40 [12], *η*_*p*_^*2*^ = .35 [13], and *η*_*p*_^*2*^ = .51 [14].

However, because these studies [12-14] did not directly compare effects of figures and scenes, we conducted the present study to investigate effects of inattention on processing of figures and scenes. Participants viewed highly arousing, negative IAPS figures and scenes and nonarousing, neutral IAPS figures and scenes at fixation and performed a visual detection task on letters that were presented either on or surrounding the pictures.

This design had two advantages compared with the design of several other, related studies. First, in other studies [5-7,12-14], the tasks requirements differed between the attended and unattended conditions (e.g., one-back task vs. letter detection task) and pictures per se were task relevant in the control task (e.g., respond to grayscale versions of the pictures). To eliminate any risk for confounds, participants in the present study performed an identical task in both conditions and the pictures were never task relevant. Specifically, participants were to respond when they detected the letter N, and attention was manipulated by presenting the target letters either on the pictures or surrounding the pictures.

Second, in other studies [12,13], the physical features of the stimuli varied slightly between the attended and unattended conditions (i.e., the number of letters between conditions). To eliminate any risk for confounds from differences in physical features of the stimuli [22], ERPs were recorded only to nontarget trials that were identical in both conditions.

In sum, one purpose of this study was to show that effects of emotion (i.e., negative vs. neutral) on EPN and LPP are larger for figures than scenes. This would suggest that negative figures are more easily understood and capture attention more strongly than do negative scenes [20]. The second purpose was to test whether inattention would reduce emotional effects on EPN and LPP less for figures than scenes. If so, then this finding would support the idea that motivated attention resists manipulations of attention more strongly for negative figures than scenes. In preview, results of the present study showed that emotional effects on EPN and LPP were larger for figures than scenes. When pictures were unattended, emotional effects on EPN increased for scenes but tended to decrease for figures, whereas emotional effects on LPP decreased similarly for figures and scenes. These findings imply that although emotional content may capture attention more strongly for figures than scenes, it does not hold it more strongly.

## Results

### Task performance

Task performance on each task (picture, letter) was indexed by d’, hit rates, and reaction time to hits. Mean d’ was greater on the picture task (M = 3.74, SD = .15) than letter task (M = 3.37, SD = .33); t(30) = 5.92, p < .001, *η*_*p*_^*2*^ = .54. Similarly, mean hit rates were greater on the picture task (M = 97.66, SD = 2.86) than letter task (M = 91.44, SD = 6.81); t(30) = 4.86, p < .001, *η*_*p*_^*2*^ = .44. In contrast, mean reaction time was shorter on the picture task (M = 507.28, SD = 54.21) than letter task (M = 592.31, SD = 73.07); t(30) = 8.17, p < .001, *η*_*p*_^*2*^ = .69. These findings showed that participants performed better and faster on the picture than letter task.

### Early posterior negativity (EPN)

**Figure 1:**
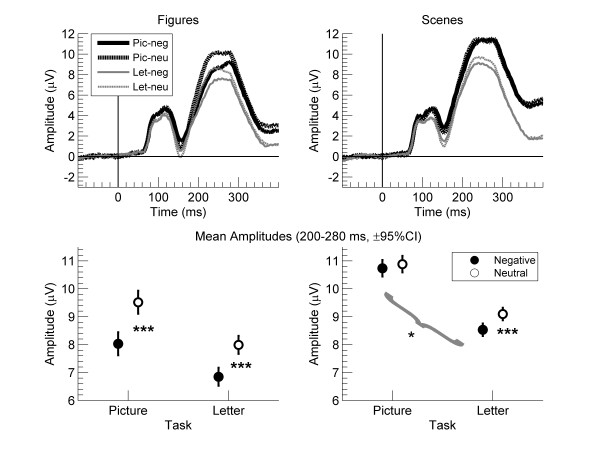
**Results for the early posterior negativity (EPN) for the letter and picture tasks for neutral and negative figures (left), and for neutral and negative scenes (right) across 12 electrodes (average referenced).** The top row shows mean ERP waves from 100 ms before to 400 ms after stimulus onset. The bottom row shows mean amplitudes across 200–280 ms. The error bars show the 95% CI of the difference scores between negative and neutral pictures in each condition, and the brackets refer to the interaction between task and emotion. ***p < .001, *p < .05

**Figure 2:**
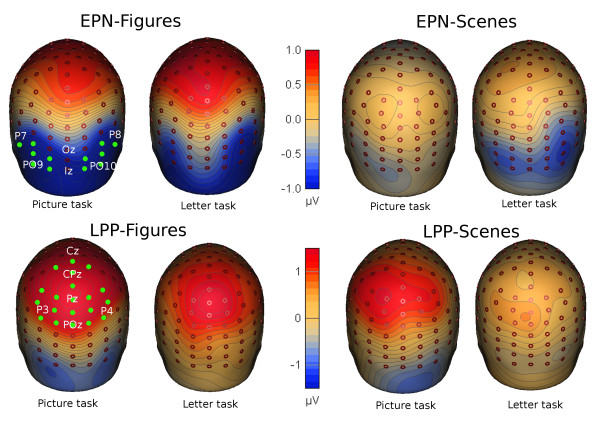
**Topographies of the main effect of emotion (negative minus neutral) for the EPN across 200–280 ms (top row) and for the LPP across 400–700 ms (bottom row).** Topographies for picture and letter tasks are shown for figures (left) and for scenes (right). The relevant electrodes (green) in computing the mean amplitudes are shown together with common 10/20 electrode positions (white)

Figures 1 and 2 show the results for the EPN. For figures (left column) and scenes (right column), the top row in Figure 1 shows mean amplitude waves between −100 and 400 ms, and the bottom row in Figure 1 shows mean amplitudes across the 200 to 280 ms interval. The top row of Figure 2 shows the corresponding topographies for figures (left column) and scenes (right column).

The repeated-measures ANOVA of mean amplitudes with task (picture, letter), composition (figure, scene), and emotion (negative, neutral) yielded main effects of task, composition, and emotion; for the main effect of task, F(1, 30) = 23.17, p < .001, *η*_*p*_^*2*^ = .44; for composition, F(1, 30) = 87.59, p < .001, *η*_*p*_^*2*^ = .75; and for emotion, F(1, 30) = 77.76, p < .001, *η*_*p*_^*2*^ = .72. As shown in Figure 1, the main effect of emotion suggested that, in general, amplitudes were less positive for negative than neutral pictures; this provides evidence for an early posterior negativity (EPN). There was also a task by composition interaction, F(1, 30) = 11.32, p = .002, *η*_*p*_^*2*^ = .27, but no task by emotion interaction, F < 1, p = .80, *η*_*p*_^*2*^ = .002. Critically, a composition by emotion interaction, F(1, 30) = 35.16, p < .001, *η*_*p*_^*2*^ = .54, showed that the amplitude difference between negative and neutral pictures was greater for figures than scenes. This effect depended on task, as indicated by the interaction of task x composition x emotion, F(1, 30) = 7.33, p = .011, *η*_*p*_^*2*^ = .20. Paired t tests showed that for scenes, amplitude differences between negative and neutral pictures increased from picture to letter task, t(30) = 2.14, p = .041, *η*_*p*_^*2*^ = .13. whereas for figures, amplitude differences tended to decrease, t(30) = 1.80, p = .082, *η*_*p*_^*2*^ = .10. Follow up paired t tests between negative and neutral pictures were significant for figures during both tasks (ts > 7.23, ps < .001, *η*_*p*_^*2*^s > .63) and for scenes during the letter task (t = 5.11, p < .001, *η*_*p*_^*2*^ = .47.) but not during the picture task (t = 1.03, p = .31, *η*_*p*_^*2*^ = .03.

Because task performance differed between tasks, an additional analysis [14] was performed to study whether differences in task performance could account for the significant three-way interaction of task x composition x emotion described above. To that end, were assigned to groups that showed either a smaller or larger change in task performance from the picture to the letter task. Then, the same ANOVA as above was performed but with the addition of the between-subjects variable of the change in task performance (median split). This allowed us to determine whether task performance moderated the task x composition x emotion interaction, that is, whether subjects who showed a larger change in task performance also showed greater effects on the task x composition x emotion interaction. Critically, there was no evidence for a four-way interaction on the basis of either d’ or hit rate (p > .89).

### Late positive potential (LPP)

**Figure 3:**
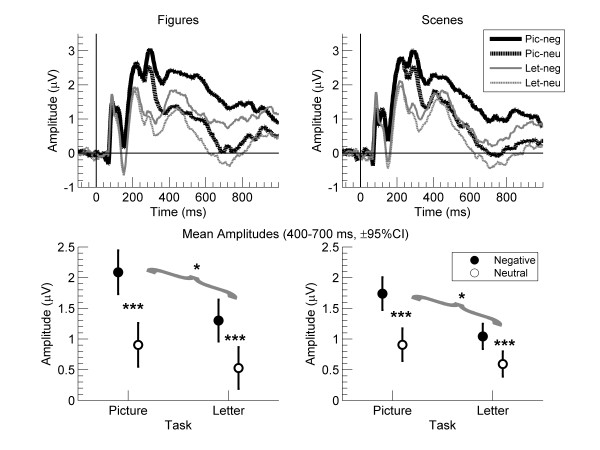
**Results for the late positive potential (LPP) for the letter and picture tasks for neutral and negative figures (left), and for neutral and negative scenes (right) across 20 electrodes (average referenced).** The top row shows mean ERP waves from 100 ms before to 1000 ms after stimulus onset. The bottom row shows mean amplitudes across 400–700 ms. The error bars show the 95% CI of the difference scores between negative and neutral pictures in each condition, and the brackets refer to the interaction between task and emotion. ***p < .001, *p < .05

Figures 2 and 3 show the results for the LPP. For figures (left column) and scenes (right column), the top row in Figure 3 shows mean amplitude waves between _100 and 1000 ms, and the bottom row in Figure 3 shows mean amplitudes across the 400 to 700 ms interval. The bottom row of Figure 2 shows the corresponding topographies for figures (left column) and scenes (right column).

The repeated-measures ANOVA of mean amplitudes with task (picture, letter), composition (figure, scene), and emotion (negative, neutral) yielded main effects of task and emotion but not of composition; for the main effect of task, F(1, 30) = 8.76, p = .006, *η*_*p*_^*2*^ = .23; for emotion, F(1, 30) = 65.80, p < .001, *η*_*p*_^*2*^ = .69; and for composition, F(1, 30) = 2.86, p = .10, *η*_*p*_^*2*^ = .09. As shown in Figure 2, the main effect of emotion suggested that, in general, amplitudes were more positive for negative than neutral pictures; this provides evidence for a late positive potential (LPP). Critically, a composition by emotion interaction showed that the amplitude difference between negative and neutral pictures was greater for figures than scenes; F(1, 30) = 6.95, p = .013, *η*_*p*_^*2*^ = .19. However, this effect was not influenced by task, as shown by an absent interaction effect for task x composition x emotion, F(1, 30) < 1, p = .88, *η*_*p*_^*2*^ = .001. But, a task by emotion interaction showed that the amplitude difference between negative and neutral pictures was greater during the picture than letter task, F(1, 30) = 7.89, p = .009, *η*_*p*_^*2*^ = .21. Paired t tests confirmed that, for both figures and scenes, amplitude differences between negative and neutral pictures decreased from picture to letter task; for figures, t(30) = 2.24, p = .033, *η*_*p*_^*2*^ = .14; and for scenes, t(30) = 2.42, p = .022, *η*_*p*_^*2*^ = .16. Also, in each task, paired t tests between negative and neutral pictures were significant for both figures and scenes (ts > 4.47, ps < .001, *η*_*p*_^*2*^ > .40).

Because task performance differed between tasks, an additional analysis [14] was performed to study whether differences in task performance could account for the significant two-way interaction of task x emotion described above. The same ANOVA as above was performed but with the addition of the between-subjects variable of the change in task performance between tasks (derived from a median split of change in task performance). Critically, there was no evidence for a three-way interaction of task x emotion x task performance (median split) on the basis of either d’ or hit rate (p > .17).

## Discussion

The main results were that across the picture task and the letter task, emotional effects (negative vs. neutral) on EPN and LPP were greater to figures than scenes. For EPN, attending the letters tended to increase emotional effects on EPN for scenes and decrease emotional effects on EPN for figures. For LPP, attending the letters decreased emotional effects on LPP similarly for figures and scenes.

Regarding the EPN (Figure 1), mean amplitudes over temporal-occipital sensors were smaller for negative than neutral pictures, that is, an EPN was evident across tasks and composition. This finding replicates reports of an EPN in many previous studies [2,3,6,7,12-14,23]. Further, the interaction of emotion by composition indicated that emotional effects on EPN were stronger for figures than scenes. This finding is consistent with the idea that emotional figures might capture attentional resources more easily than do emotional scenes [20].

For EPN, effects of task on emotion varied for figures and scenes, as indicated by a significant three way interaction between emotion, composition, and task. However, the pattern of results was counterintuitive. For figures, emotional effects on EPN were present during both tasks with only a trend to decrease from the picture to the letter task. For scenes, emotional effects on EPN were absent during the picture task but present during the letter task. Thus, the three way interaction suggested that for scenes, emotional effects on EPN increased from the picture to the letter task. This result is puzzling for two reasons: First, previous research demonstrated clear emotional effects on EPN when participants attended the pictures [2,3,6,7,12-14,23]. Second, previous research showed that when participants did not attend the pictures, emotional effects on EPN either were unaffected [7,12,13] or decreased [6,14]. Therefore, the direction of the three way interaction is opposite to previous research and does not support our hypothesis that inattention reduces emotional effects on EPN less for figures than scenes.

Regarding the LPP (Figure 2), results showed that mean amplitudes over central parietal sensors were greater for negative than neutral pictures, that is, an LPP [24] was evident across tasks and composition. As indicated by the emotion by composition interaction, emotional effects on LPP were stronger for figures than scenes. These findings replicate previous findings and support the idea that the emotional content in figures might capture attentional resources more easily than does the emotional content in scenes [20]. Further, results showed an interaction between emotion and task. Emotional effects on LPP decreased when participants attended the letters, replicating previous findings [5,6,8,9,12,14].

Critically, there was no evidence that task effects on emotion varied for figures and scenes. Thus, the nonsignificant three-way interaction between emotion, composition, and task suggested that emotional effects on LPP to figures and scenes decreased similarly when letters rather than pictures were attended. So, even if negative versus neutral pictures elicited stronger emotional effects on LPP for figures than scenes, this response was not more resistant to manipulations of attention. Notably, neither EPN nor LPP were eliminated when pictures were unattended. This corroborates findings that EPN and LPP to unattended negative figure-ground pictures at fixation are not eliminated easily [10-13].

Finally, we note four caveats. First, our picture set was limited to negative and neutral pictures and therefore, it is unresolved whether our results apply to pictures of positive valence. But, instead of selecting fewer pictures from all three valence categories, we broadly selected pictures from a wide range of negative emotions to be able to generalize our results to negative valence [15].

Second, our picture set comprised negative and neutral pictures and was not divided further into different discrete emotions (e.g., anger, fear, disgust) or picture types (e.g., face, snake, etc.). Although future research needs to determine whether discrete emotions and picture types differ in their effects, there are two challenges: On one hand, discrete emotions and picture types often differ in valence and arousal ratings; thus, it is difficult to rule out the possibility that different effects may be caused simply by differences in valence and arousal [25]. On the other hand, it is difficult to find enough individual IAPS pictures that elicit only a single discrete emotion because most IAPS pictures evoke a mixture of emotions [26].

Third, although both tasks showed clear ceiling effects (hit rates > 90%), there was a slight but significant performance difference between the picture task and the letter task. Although this small performance difference is an unlikely confound [13,14], we computed, for each subject, the difference in task performance between the two tasks and grouped subjects according to a median split. Critically, the effects of task on the EPN (i.e., the three-way interaction between task, composition, and emotion) and on the LPP (i.e., the two-way interaction between task and emotion) were not moderated by the change in task performance. These results suggest that the task effects on EPN and LPP were not caused by performance differences between tasks.

Fourth, the present study did not collect data on individual differences such as trait anxiety and depression. Therefore, an important question for future research will be to study any potentially moderating effects of individual differences on the ERP responses.

## Conclusions

The present results replicate and extend previous reports that effects of emotion (negative vs. neutral) on EPN and LPP are larger for figures than scenes. Together, these findings support the idea that because figures are simpler than scenes, the emotional content in figures captures attention more strongly than does the emotional content in scenes [20]. Generally put, because picture composition is simpler for figures than scenes, the content of the pictures is extracted more easily from figures than scenes. As a consequence, the emotional content is also extracted more easily from figures than scenes and thus, the emotional content has a stronger effect on attention for figures than scenes. Accordingly, the emotional content in figures elicits a stronger call for attentional resources (indexed by EPN) and a stronger allocation of attentional resources (indexed by LPP) than does the emotional content in scenes. However, our results showed that when participants attended the letters rather than the pictures, emotional effects on EPN and LPP decreased similarly for figures and scenes. These findings suggest that the emotional content does not hold attention more strongly for figures than scenes. So, compared to emotional scenes, emotional figures elicit a stronger call for attentional resources and a stronger allocation of attentional resources when pictures are attended. However, these effects on attention are reduced similarly for figures as for scenes when pictures are unattended.

## Methods

### Participants

Thirty-one participants (16 women) with a mean age of 27 years (SD = 8.39, range 18–50) were recruited. The research was approved by the regional ethics board. Participation was based on informed consent and was rewarded with course credits or movie vouchers.

### Stimuli

Negative (n = 100) and neutral (n = 100) color pictures in landscape without black frames (1024 x 768 pixels), were selected from the International Affective Picture System (IAPS) [18]. The pictures were selected on the basis of standardized pleasure and arousal ratings and of picture composition ratings obtained in a pilot study with 9 participants. The task of rating picture composition was modeled after that in Bradley et al. [20]. Accordingly, composition ratings were obtained on a scale between 1 and 9 where 1 indicated that the picture had a clear figure-ground composition (figures) and 9 indicated that the picture was a complex scene (scenes). Participants were instructed to ignore the emotional meaning of the pictures. Before the actual task, they were provided with examples of negative and neutral figures and scenes.

Pictures were selected so that the negative and neutral picture sets each comprised equal numbers (n = 50) of figures and scenes. Mean composition ratings were 2.53 (SD = .54) for negative figures, 2.27 (SD = .69) for neutral figures, 5.34 (SD = 1.08) for negative scenes, and 5.42 (SD = 1.33) for neutral scenes. In an ANOVA of mean composition ratings with emotion (negative, neutral) and composition (figures, scenes), the ANOVA showed only a main effect of composition, F(1, 196) = 478.81, p < .001, but no main effect of emotion, F(1, 196) < 1, p > .50, and no interaction between emotion and composition, F(1, 196) = 1.50, p = .22. Mean normative valence ratings were 2.58 (SD = .80) for negative figures, 5.21 (SD = .53) for neutral figures, 2.53 (SD = .63) for negative scenes, and 5.14 (SD = .59) for neutral scenes. In an ANOVA of mean valence ratings with emotion (negative, neutral) and composition (figures, scenes), the ANOVA showed only a main effect of emotion, F(1, 196) = 826.88, p < .001, but no main effect of composition, F(1, 196) < 1, p > .50, and no interaction between emotion and composition, F(1, 196) < 1, p > .50. Mean normative arousal ratings were 6.24 (SD = .57) for negative figures, 3.48 (SD = .33) for neutral figures, 6.32 (SD = .40) for negative scenes, and 3.49 (SD = .35) for neutral scenes. In an ANOVA of mean arousal ratings with emotion (negative, neutral) and composition (figures, scenes), the ANOVA showed only a main effect of emotion, F(1, 196) = 2144.86, p < .001, but no main effect of composition, F(1, 196) < 1, p = .45, and no interaction between emotion and composition, F(1, 196) < 1, p > .50. Thus, for negative and neutral pictures, mean pleasure and arousal ratings did not differ between figures and scenes.

**Figure 4:**
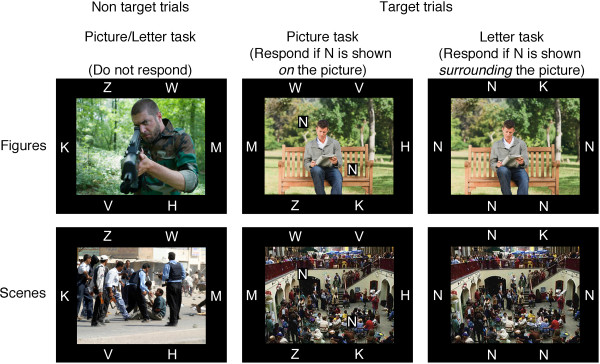
**Illustration of the task stimuli and instructions.** Non-target trials for both tasks (left panel), and target trials in the picture task (middle panel) and letter task (right panel). ERP data were collected only during non-target trials. These trials consisted of a picture with either a simple figure ground composition (top row) or a complex scene composition (bottom row) that was surrounded by six distracter letters. In the picture task, participants responded when the letter N was shown on the picture. In the letter task, participants responded when the letter N replaced five of the six distracters

Negative pictures included attacking animals, mutilated bodies, guns, and angry faces; neutral pictures included animals, household objects, tools, plants and neutral faces. The picture list can be obtained on request. These pictures were either figures or scenes where an example of a negative figure was a person holding a gun, a negative scene was a group of people holding guns during a riot, a neutral figure was a person sitting on a bench, and a neutral scene was a group of people in a marketplace (Figure 4).

Pictures were presented on a 21-inch Viewsonic P227f CRT monitor at 1024 × 768 resolution at a refresh rate of 100 Hz. The viewing distance was held constant at 80 cm with a chin rest. The experimental presentation was programmed and executed in Presentation software (Neurobehavioral Systems).

### Procedure

Each trial began with a fixation cross at the center of the screen (randomly at 800, 900 or 1000 ms) whereafter an IAPS picture was shown for 200 ms. Pictures (13.2° × 9.3°) were shown at the center of the screen surrounded by six distracter letters (Figure 4). The distracter letters were bordering the pictures in 6 locations; 2 each above and below at 5.7° and 3.2° left and right from fixation, and 1 each left and right at 7.5° from fixation. Distracter letters were H, K, M, V, W and Z (1.1° tall, 0.8–1.4° wide). Participants were instructed to press the space key (within 1300 ms) whenever the target letter (N) was shown (20% of trials). On target trials of the picture task, two Ns were shown on the picture in random locations varying from 1.1° to 3.9° above or below and 1.1° to 3.9° left or right. The two Ns were shown in opposite quadrants to minimize reflexive saccades. So, if the left N was shown in the upper quadrant, the right N was shown in the lower quadrant (or vice versa). On target trials of the letter task, five Ns replaced five of the six distracter letters (in random locations).

Each task was presented twice in separate blocks. Each block consisted of 200 trials, and all 200 pictures were shown once. Trial order was random, and task order was counterbalanced across participants (i.e., ABBA). Before the first block of each task, 20 practice trials were administered to familiarize participants with the procedure. Pictures were the same in both practice tasks and were not reused during the actual experiment. Participants were instructed to keep their gaze at the center of the screen (at the position of the fixation cross) throughout the task and to respond as quickly as possible while minimizing errors.

### Physiological recording

Electroencephalography (EEG) was recorded with an ActiveTwo system (BioSemi, Amsterdam, Netherlands) from 128 sites at 512 Hz sampling rate and filtered only with a build-in low pass filter at 104 Hz and an offline notch filter at 50 Hz. Electrodes were mounted in an elastic cap and arranged according to the ABC system (i.e., electrodes are arranged in concentric rings with different distances from the vertex, http://www.biosemi.com/headcap.htm).

### Data reduction and analysis

The software BESA (Version 5.3.7, BESA GmbH, Gräfelfing, Germany) was used for offline processing of the EEG. Built-in ocular artifact-detection and correction algorithms (15 surrogate brain sources) were applied to the raw EEG signal. Noisy electrodes (maximum of 3 for 4 participants) were interpolated with spherical splines. To reduce confounding effects from motor responses, only non-target trials without button presses were analyzed. EEG epochs were created for each trial extending from 100 ms before to 1000 ms after stimulus onset with a 100-ms baseline. All EEG data were re-referenced to the arithmetic average of all 128 electrodes.

To identify clusters of electrodes and intervals corresponding to the EPN and LPP, ERP difference waves to the negative minus neutral pictures were inspected visually. ERPs were collapsed across participants and tasks to detect a main effect of emotion (i.e. negative minus neutral). Thus, EPN and LPP were defined as the difference waves between negative and neutral pictures. A greater negativity to aversive versus neutral pictures (i.e. EPN) was evident between 200 and 280 ms across 12 electrodes (A10-14, A26-27, B07-10, D32), and a greater positivity to aversive versus neutral pictures (i.e., LPP) was strongest between 400 and 700 ms across 20 electrodes (A02-08, A17-21, A30-32, B02-05, D16). These electrodes and intervals for EPN and LPP matched that of previous studies in our lab [12-14]. For each participant, the mean amplitudes (in μV) for the relevant electrodes and intervals were extracted for each trial and then averaged separately for each block, task, composition, and emotion to create a grand average for each factor used in the ANOVA. Because in preliminary analyses, gender and block had no effects on the amplitudes of the ERPs, the simpler ANOVAs are reported.

In an additional analysis, we extracted P1 amplitudes. The P1 was apparent across all negative and neutral pictures between 90 and 150 ms across 18 electrodes (A09-11, A14-16, A27-29, B06-08, B10-12, D30-32). Results of the ANOVA showed only a main effect of task, F(1, 30) = 32.41, p < .001, *η*_*p*_^*2*^ = .52. Mean P1 amplitudes were greater during the picture task (M = 3.88) than the letter task (M = 3.14). Critically, all effects involving emotion were not significant.

Last, in regards to behavioral analysis, on target trials (i.e., response trials) during the picture task, the letters were superimposed on the pictures. Because effects of the superimposed letters might vary for emotion and composition, the analysis of behavioral data included only effects of task to obtain a general measure of task performance.

## Endnotes

^a^This study reported the positive pole of the EPN over central electrodes. Whereas the EPN is commonly measured as a negativity to emotional versus neutral pictures over occipital electrodes, an alternative approach is to measure the polarity reversal over central electrodes [2,4].

## Authors’ contributions

Both authors contributed equally to the study.
